# Calcification of the Internal Carotid Artery and Its Influence on the Severity of Cerebral Vasospasm in Aneurysmal Subarachnoid Hemorrhage

**DOI:** 10.3390/jcm15010168

**Published:** 2025-12-25

**Authors:** Adrian Engel, Laurèl Rauschenbach, Argtim Rexhepi, Meltem Gümüs, Christoph Rieß, Jan Rodemerk, Li Song, Yan Li, Börge Schmidt, Yahya Ahmadipour, Philipp Dammann, Marvin Darkwah Oppong, Ulrich Sure, Ramazan Jabbarli

**Affiliations:** 1 Department of Neurosurgery and Spine Surgery, University Hospital Essen, 45147 Essen, Germany; 2 Center for Translational Neuroscience and Behavioral Science (C-TNBS), University of Duisburg-Essen, 47057 Essen, Germany; 3 Institute for Diagnostic and Interventional Radiology, University Hospital Essen, 45147 Essen, Germany; 4 Institute for Medical Informatics, Biometry and Epidemiology, University Hospital Essen, 45147 Essen, Germany

**Keywords:** subarachnoid hemorrhage, cerebral vasospasm, atherosclerosis

## Abstract

**Background/Objectives:** Cerebral vasospasm (CV) is a serious complication of aneurysmal subarachnoid hemorrhage (aSAH). Carotid siphon calcification (CSC) has been associated with a reduced risk of CV. This study investigates the influence of CSC on the clinical and radiographic severity of CV and functional outcome of aSAH. **Methods:** A total of 475 patients with aSAH treated at the University Hospital Essen (2008–2016) were analyzed retrospectively. CSC was assessed using the Woodcock score. Study endpoints were the CV severity in digital subtraction angiography, presence of CV in transcranial Doppler (TCD) ultra-sonography, occurrence of delayed ischemic neurological deficit (DIND) and the functional outcome at 6 months measured with the modified Rankin scale. **Results:** CSC was confirmed as an independent predictor for the occurrence (aOR 0.76; 95% CI 0.60–0.97; *p* = 0.025) and severity (RC −0.14; 95% CI −0.24 to −0.04; *p* = 0.006) of angiographic CV and development of DIND (aOR 0.76; 95% CI 0.59–0.98; *p* = 0.034). Only the duration (in days: RC −0.43; 95% CI −0.77 to −0.10; *p* = 0.010) but not the presence (aOR 0.87; 95% CI 0.68 to 1.11; *p* = 0.265) and severity (cerebral blood flow, in cm/s: RC +1.57; 95% CI −7.45 to +10.58; *p* = 0.731) of TCD CV was associated with CSC. Finally, the increasing levels of CSC were related to poorer 6-month functional outcome (RC +0.12; 95% CI +0.05 to +0.18; *p* < 0.001). **Conclusions:** CSC appears to be protective against angiographic CV and DIND, but correlates with worse overall outcome, suggesting that atherosclerosis, represented by CSC, affects cerebrovascular regulation and overall prognosis. We suggest careful evaluation of primary imaging studies for markers of atherosclerosis to identify patients at risk for CV and patients with low risk for CV but still at high risk for poor outcome.

## 1. Introduction

In the treatment of aneurysmal subarachnoid hemorrhage (aSAH), cerebral vasospasm (CV) is a feared secondary complication [1]. Within the literature, the early detection and aggressive endovascular treatment of CV are known to reduce the risk of delayed cerebral ischemia (DCI) and to improve the functional neurological outcome [2]. Clinical and radiologic bleeding severity, smoking, female sex, and young age have been described as risk factors for CV [3,4]. Conversely, carotid calcification and intracranial calcification have been shown to be protective against angiographic and Doppler-sonographic CV [5,6,7]. However, carotid siphon calcification (CSC) was not only protective against angiographic CV but was also associated with an increased risk of DCI and an impaired functional neurological outcome [8].

CSC is linked with carotid stenosis and atherosclerosis [9]. Older age and a higher burden of atherosclerosis are associated with an increased arterial stiffness, which disrupts cerebral blood flow regulation [10,11]. Although CSC decreases the risk of CV after aSAH, at the same time it increases DCI risk due to disruption of the cerebral blood flow (CBF) [8]. Moreover, the influence of the CSC on clinical and radiographic severity and duration of CV has not been analyzed.

In this study, using a large consecutive aSAH cohort, we aimed to assess the impact of CSC on the severity of angiographic CV, the characteristics of CV detected through daily transcranial Doppler (TCD) ultrasonography, and the clinical manifestations of CV, specifically the occurrence of delayed ischemic neurological deficits (DINDs). Additionally, we evaluated the influence of CSC on long-term functional outcomes after aSAH.

## 2. Materials and Methods

From September 2008 to June 2016, 498 consecutive patients were included in our institutional aneurysm database at the university hospital of Essen. Patients with an aSAH and a CT scan available in our image viewing system were included in this retrospective study (*n* = 475). The local ethics committee approved this study, with the registration number 15-6331-BO. All patients or their legal representatives gave their written consent for the inclusion into the aneurysm database. The study is registered in the German Register for Clinical Trials (unique identifier: DRKS00008749; date of approval 30 June 2015, date of approval of supplementary requests 18 October 2016, 5 February 2019, 12 November 2019, 10 December 2019). The study data is available from the corresponding author upon reasonable request.

### 2.1. Treatment Process

All patients with suspected aSAH were subjected to a computed tomography (CT) scan upon arrival, unless a definitive diagnosis had already been established at another hospital. In such cases, the latter was the secondary referral center for the patients to our tertiary care center. Following the confirmation of the diagnosis, patients were subjected to digital subtraction angiography (DSA) in order to determine the source of bleeding. In instances where an aneurysm was identified as the bleeding source, the optimal course of action was discussed in a multidisciplinary fashion, including the possibility of performing either endovascular or microsurgical aneurysm occlusion. Patients were then transferred to our neurosurgical intensive care unit, where they remained for at least 14 days. Daily TCD studies were performed, and the patients were closely neurologically monitored to detect sings of possible CV. Oral or intravenous nimodipine was administered prophylactically to all aSAH patients during 3 weeks after aSAH. Acute hydrocephalus was managed with an external ventricular drain allowing continuous intracranial pressure (ICP) monitoring and drainage of cerebrospinal fluid if necessary. In case of elevated ICP >20 mmHg, medical management was performed to lower the ICP, followed by decompressive craniectomy in individuals with ICP crisis refractory to conservative management.

Events of new onset of focal or global neurological deficit, that could not be linked to other events, like, e.g., ICP increase, seizure, or rebleeding, were regarded as DINDs attributable to CV [12]. In these cases, the patients were taken for repeated DSA for further CV management. Moreover, DSA for CV verification was also initiated in unconscious aSAH patients with increasing cerebral blood flow velocity ([CBFV] mean value > 120 cm/s) in TCD. Confirmed angiographic CV was treated with intra-arterial nimodipine and, if insufficient, with angioplasty. After the acute monitoring period and hospitalization, all patients were transferred to appropriate neurological rehabilitation units. Patients were followed up clinically at our outpatient service, or, if not possible, by telephone at 6 months after aSAH.

### 2.2. Data Management and Statistics

**Figure 1:**
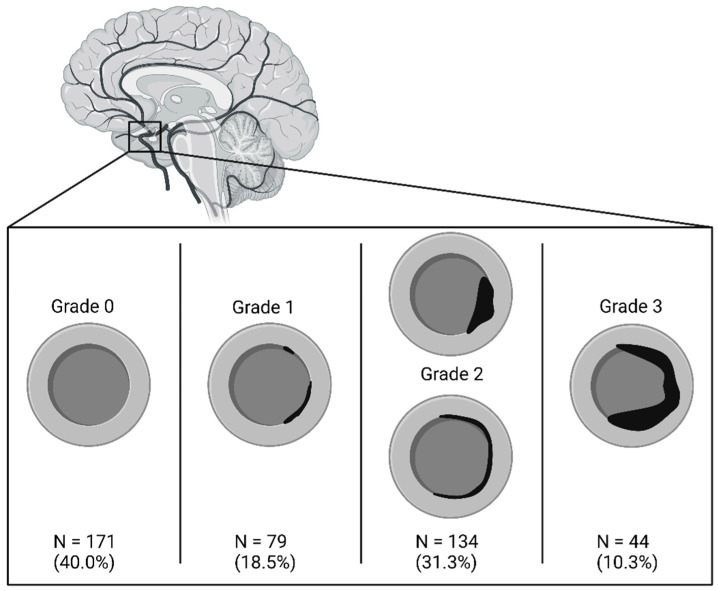
An illustration of the Woodcock scale: grade 0: no calcification; grade 1: thin discontinuous calcification; grade 2: thin continuous calcification or thick discontinuous calcification; grade 3: thick continuous calcification [13]. Created in BioRender.com.

**Figure 2:**
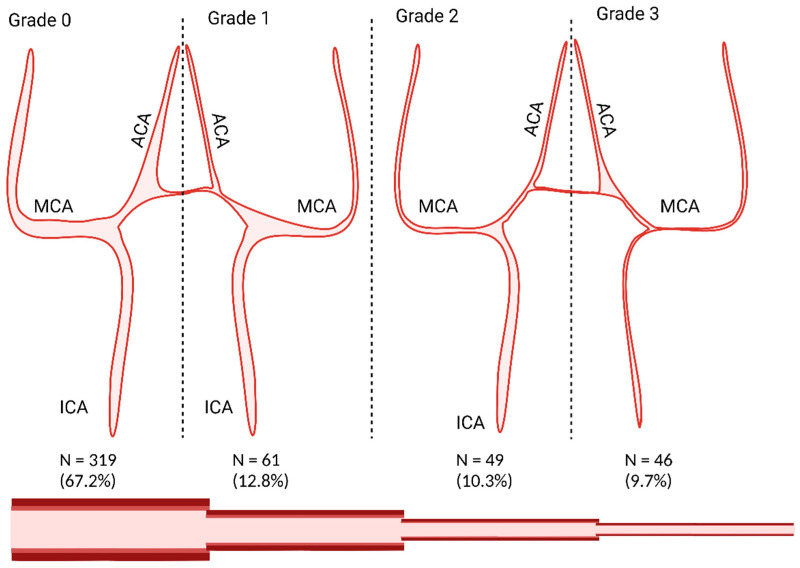
An illustration of the Merkel scale using following grades: grade 0: all intracranial vessels show a physiological shape; grade 1: minimal narrowing affecting A2, A1 and/or M2; grade 2: moderate narrowing from M1 and terminal (intradural) segments of the internal carotid artery; grade 3: severe narrowing of the intradural internal carotid artery and the M1 with filiform (“ghost-like”) appearance of A1 and M1) [14]. Created in BioRender.com.

The objective of this study was to analyze the impact of the CSC on the severity of the CV. To quantify the findings, the first author (AE) evaluated the non-contrast-enhanced CT scans and graded the most severe CSC for each patient according to the Woodcock score (grade 0: no calcification; grade 1: thin discontinuous calcification; grade 2: thin continuous calcification or thick discontinuous calcification; grade 3: thick continuous calcification) [13]; see Figure 1. To quantify the CV, the first author analyzed all available DSAs and graded the most severe CV on the DSAs according to the scale proposed by Merkel et al. (grade 0: all intracranial vessels show a physiological shape; grade 1: minimal narrowing affecting A2, A1 and/or M2; grade 2: moderate narrowing from M1 and terminal (intradural) segments of the internal carotid artery; grade 3: severe narrowing of the intradural internal carotid artery and the M1 with filiform (“ghost-like”) appearance of A1 and M1) [14]; see Figure 2. If no DSA was performed, the angiographic grade was defined as 0, and CV was considered absent.

The electronic health records of patients and the institutional aneurysm database were screened for the following data: clinical (using the World Federation of Neurosurgical Societies [WFNS] scale [15]) and radiographic (using the original Fisher scale [4]) bleeding severity, presence of acute hydrocephalus, type of aneurysm treatment (endovascular or microsurgical), daily TCD measurements, occurrence of ICP increase >20 mmHg, and/or DIND. Additionally, the following acknowledged or widely disputed risk factors for CV were also recorded for further analysis as confounders: age, sex, and smoking status. In all patients, the clinical outcome was documented 6 months after the discharge from hospital using the modified ranking scale (mRS) [16].

A descriptive statistic was performed, using mean values (± standard deviation [SD]), median values (interquartile range [IQR]), or absolute numbers (relative amount [%]), wherever applicable. A binary logistic regression was used to predict the occurrence of angiographic and TCD CV and DIND, adjusting for the risk factors such as age, sex, smoking status, radiographic (fisher score ≥ 3) and clinical (WNFS score ≥ 4) bleeding severity, treatment modality, and ICP crisis. Results were presented using adjusted odds ratios (aOR) and the 95% confidence interval (CI), as well as *p*-value (≤0.05). Additionally, a multinominal linear regression was performed to analyze the impact of CSC on the severity of angiographic CV, the number of days with increased CBFV, and the maximal CBFV values in TCD, using the same confounders, as mentioned above. Results are preesnted using regression coefficient (RC) and 95% CI, as well as *p*-value (≤0.05). Missing values were handled by multiple imputations for the following variables: patient age, patient sex, smoking status, Fisher grade, WFNS grade, aneurysm occlusion, angiographic CV, increased TCD, DIND, severity of angiographic CV (according to the Merkel scale), days of increased TCD values (>120 cm/s), and mRS 6 months after discharge. Additionally, a multinominal linear regression was performed to predict the outcome 6 months after discharge (using the mRS) based on the maximum CSC per patient, using the same confounders as mentioned above and also including the severity of the angiographic CV as a confounder. In all performed analyses, CSC was used as a continuous variable.

Statistical analysis was performed using IBM SPSS Statistics Version 29.0.0.0, and GraphPad PRISM Version 9.0 was used for visualization. All correlations with *p* ≤ 0.05 were considered significant. www.BioRender.com was used for visualization of the Woodcock scale and the Merkel scale. The manuscript was checked and improved by using www.DeepL.com. The article follows the STROBE reporting guidelines.

## 3. Results

The mean age of the final study population (*n* = 475) was 55.83 years (±SD 14.04), 320 patients were female (67.4%), and 267 patients were microsurgically clipped (58.4%). The missing values are displayed in absolute numbers. For detailed information on the study population, see Table 1.

There was no CSC in 40% of the cohort, and 41.6% had a CSC grade 2 or higher; see Figure 1. The distribution of angiographic severity of CV is shown in Figure 2. Approximately two-thirds of the aSAH population did not experience any CV. In total, 20% of the patients had a CV grade 2 or higher.

**Figure 3:**
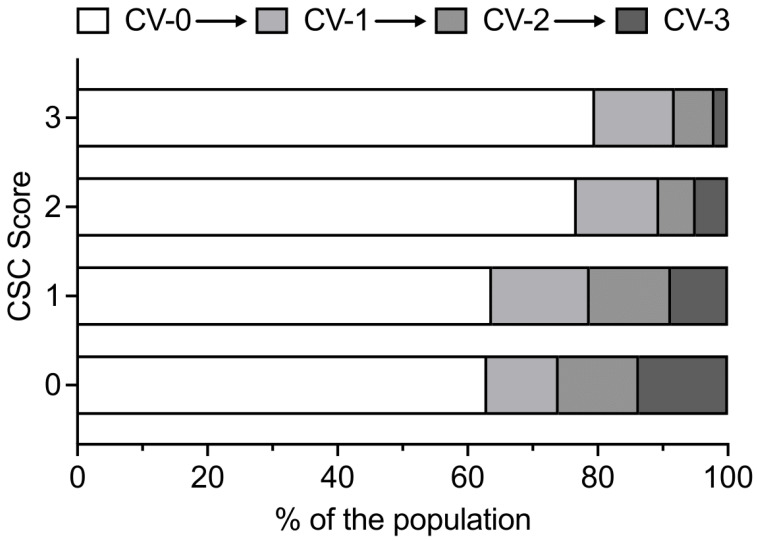
Bar chart displaying the different relative amounts of severities of angiographic CV (using the scale by Merkel et al. [14]) for the different grades of CSC (using the scale by Woodcock et al. [13]). This shows a decrease in the severity of CV with increasing CSC levels. Abbreviations: carotid siphon calcification (CSC); cerebral vasospasm (CV).

Within the binary logistic regression, the CSC showed independent association with the occurrence of DIND (aOR 0.76; 95% CI 0.59–0.98; *p* = 0.034) and angiographic CV (aOR 0.76; 95% CI 0.60–0.97; *p* = 0.025); see Table 2. Additionally, CSC was predictive of the severity of the angiographic CV (RC −0.14; 95% CI −0.24 to −0.04; *p* = 0.006) in the linear regression; see Table 3. The distribution of angiographic CV severity in the cohort depending on the CSC gradings is shown in Figure 3. A decline in the relative proportions of higher-grade CVs has been observed, concomitant with an increase in CSC.

With regard to the manifestation of CV in TCD observations, multivariable analysis did not demonstrate a significant correlation between CSC severity and the occurrence of CV (aOR 0.87; 95% CI 0.68–1.11; *p* = 0.265; see Table 2), nor with the absolute maximum CBFV value (RC +1.57; 95% CI −7.45 to +10.58; *p* = 0.731; see Table 3). Nevertheless, a substantial inverse relationship was observed between CSC severity and the number of days on which CVs (>120 cm/s) were recorded by TCD. (RC −0.43; 95% CI −0.77 to −0.10; *p* = 0.010; see Table 3).

Conversely, a higher maximum severity of CSC has been shown to correlate with poorer functional outcomes at six months following aSAH (RC +0.12; 95% CI +0.05 to +0.18; *p* < 0.001); see Table 4.

## 4. Discussion

Our data shows that atherosclerosis of the large intracranial vessels, as represented by CSC, not only has a protective effect against the occurrence of CV, but also mitigates the severity of angiographic CV.

Specifically, we observed that higher levels of CSC were associated with lower rates of angiographic CV. Furthermore, our findings reveal an inverse correlation between increasing CSC levels and the severity of angiographic CV, suggesting a potential protective mechanism. A previous study performed by our group [8] showed an inverse correlation between the occurrence of an angiographic CV in the specific vascular territories of ICA, ACA, and MCA and the CSC, also measured using the Woodcock scale [13]. This protective effect of CSC against CV could be caused by the increasing arterial stiffness in patients with a higher level of atherosclerosis [10,11]. Our data suggests that any level of atherosclerosis in the cerebral vessels reduces the severity of angiographic CV. These findings contradict the results of previous studies, which reported a protective effect against CV detected by TCD only at higher levels of atherosclerosis [6]. Interestingly, the CSC was found to be independently linked to a decreased severity of angiographic CV, even though the analyses included age as a confounder. It is generally accepted that younger age is one of the major risk factors for suffering from CV [3], while older age is known to be a risk factor for the progression of atherosclerosis [17]. This observation, when supported by these findings, suggests that younger age itself may not be a significant risk factor and may merely coincide with more severe CV, most likely due to a general lower prevalence of atherosclerosis, such as CSC, in this age group. At the same time, our analysis confirms age as a significant risk factor for both the occurrence and severity of CV, indicating that its influence may extend beyond the effects of atherosclerosis alone.

Although our data showed a predictive value of the CSC for angiographic CV, we could not predict CV detected by TCD using a cut-off value of >120 cm/s. Another study showed an inverse correlation between the CSC and the occurrence of TCD-detected CV, but also an inverse correlation between CSC and the severity of TCD-detected CV [7]. Our data did not show these inverse correlations. It is possible that vessel reactivity is decreased by intracranial atherosclerosis. This hypothesis is supported by the evidence that such atherosclerosis has been demonstrated to reduce cerebral autoregulatory responses to neuronal activity and the CBF [11]. Therefore, it may be that lower levels of CV are not as readily detected by TCD, which naturally covers only the larger intracranial vessels, in patients with higher levels of CSC. A different cut-off threshold might be chosen in future studies to further investigate this hypothesis. However, due to a loss of data, we were not able to analyze the TCD-measured CV utilizing different cut-offs.

In addition, we were able to show a lower probability of DIND in aSAH individuals with higher level of CSC. A correlation between the occurrence of DIND and CV has been reported in the literature [12,18], supporting our findings of a lower incidence of DIND in patients with CSC. However, it has also been critically discussed that angiographic CV is not exclusively the cause of DIND and that it could also be caused by local changes in cerebral perfusion [19]. This association is not explained by our data but could be related to local small CV not detected by either TCD or DSA, and it opens new questions for future research projects.

However, our data also showed a link between higher levels of CSC and unfavorable outcomes, independent of the severity of the angiographic CV. This clearly shows that the protective effect against CV, be it angiographic or Doppler sonographic, and the protective effect against DIND are not the only factors influenced by the higher burden of atherosclerosis shown by CSC. CSC has been shown to be predictive of the occurrence of early cerebral ischemia, but not of delayed cerebral ischemia [8]. The missing link to the DCI is still unexplained and can only be postulated due to a multifactorial process leading to these DCIs and therefore potentially leading to an unfavorable outcome. Potential effects leading to worse neurological outcomes could be due to the generally increased incidence of ischemic events in populations with a higher burden of atherosclerosis [20,21]. The higher burden of cerebral small vessel disease [22] and the higher burden of downstream micro emboli in patients with higher levels of atherosclerosis [23] may also contribute. Furthermore, the lack of appropriate CBF responses to neural activities [11] could be a contributing factor to fill be the missing link between the decreased severity of CV at higher levels of CSC.

The limitations of this study are its purely retrospective nature and therefore its selection bias. Nonetheless, the implementation of linear and binary logistic regression, in conjunction with the correction of analyses for the aforementioned confounders, has been demonstrated to partially surmount these limitations. Furthermore, the results of the lack of correlation with CBFV should be interpreted with caution due to the high number of missing patients in this analysis. Additionally, the calcifications and plaques in the carotid artery were not differentiated, and potentially vulnerable non-calcified plaques were not analyzed separately.

## 5. Conclusions

In summary, our data strongly suggests that CSC not only protects against the occurrence of angiographic CV, but also reduces the severity of angiographic CV, reduces the duration of CV detected by TCD, and reduces the incidence of DIND in aSAH patients. Our findings indicate that the atherosclerotic status of patients, as measured by CSC, should be incorporated into patient therapy. This would enable the identification of patients at risk of CV with lower CSC levels, as well as patients with lower CV risk who are still at higher risk of adverse outcomes.

## Figures and Tables

**Table 1:** Descriptive statistics of the used confounders and populations characteristics using the mean (±SD), median (IQR), or total amount (relative amount %), whenever appropriate. Abbreviations: standard deviation (SD); interquartile range (IQR); cerebral vasospasm (CV); cerebral blood flow velocity (CBFV); transcranial Doppler (TCD); carotid siphon calcification (CSC); World Federation of Neurosurgical Societies (WFNS); intracranial pressure (ICP).

	Mean (±SD), Median (IQR) or Total Amount (Relative Amount)
Maximum CSC value	1 (0–2)
Patient age	55.83 (±14.04)
Patient sex (female)	320 (67.4%)
Smoking status (yes)	155 (32.6%)
Fisher grade (3 or 4)	391 (86.1%)
WFNS grade (4 or 5)	195 (41.1%)
Aneurysm occlusion (microsurgical)	267 (56.2%)
ICP crisis (>20 mmHg)	152 (32.3%)
Maximum angiographic CV per patient	0 (0–1)
Increased CBFV (>120 cm/s)	216 (48.4%)
Days of increased CBFV (>120 cm/s)	2.33 (±3.39)
Maximum CBFV by TCD	158.53 (±50.68)

**Table 2:** Binary logistic regression for the prediction of angiographic CV, Doppler-sonographic CV and DIND, with the following confounders: maximum CSC per patient (using the scale by Woodcock et al. [13]), patient age, patient sex, smoking status, Fisher scale (dichotomized for ≥3 and <3), WFNS scale (dichotomized for ≥4 and <4), type of aneurysm occlusion (microsurgical vs. endovascular), and ICP crisis (>20 mmHg) as confounders. The results are displayed using aOR (95% CI) and *p*-values. Abbreviations: cerebral vasospasm (CV); transcranial Doppler (TCD); delayed ischemic neurologic deficit (DIND); adjusted odds ratio (aOR); confidence interval (CI); carotid siphon calcification (CSC); World Federation of Neurosurgical Societies (WFNS); intracranial pressure (ICP).

	Angiographic CV Present		Increased TCD Values (>120 cm/s)		DIND	
	aOR (95% CI)	*p*-Value	aOR (95% CI)	*p*-Value	aOR (95% CI)	*p*-Value
CSC, per-point increase	0.76 (0.60–0.97)	0.025	0.87 (0.68–1.11)	0.265	0.76 (0.59–0.98)	0.034
Patient age, per-year increase	0.98 (0.96–1.00)	0.030	0.95 (0.94–0.97)	<0.001	1.01 (0.99–1.02)	0.615
Patient sex (female)	1.16 (0.75–1.81)	0.503	1.07 (0.68–1.69)	0.768	1.06 (0.67–1.68)	0.816
Smoking status (yes)	0.56 (0.35–0.88)	0.012	0.54 (0.34–0.86)	0.009	0.70 (0.43–1.15)	0.157
Fisher grade (3 or 4)	1.96 (0.98–3.91)	0.057	2.03 (1.07–3.87)	0.031	1.22 (0.59–2.56)	0.587
WFNS grade (4 or 5)	0.73 (0.45–1.16)	0.183	0.88 (0.54–1.44)	0.612	0.84 (0.51–1.37)	0.477
Aneurysm occlusion (microsurgical)	1.61 (1.04–2.49)	0.035	1.66 (1.06–2.61)	0.028	0.92 (0.57–1.47)	0.719
ICP crisis (>20 mmHg)	1.92 (1.19–3.11)	0.008	2.85 (1.72–4.72)	<0.001	1.94 (1.16–3.23)	0.010

**Table 3:** Multinominal linear regression for the prediction of the angiographic grade of the CV, using the scale of Merkel et al. [14], the severity of Doppler sonographic CV (measured by the maximum CBFV) and the days of increased TCD values (>120 cm/s), with the following confounders: maximum CSC per patient (using the scale by Woodcock et al. [13]), patient age, patient sex, smoking status, Fisher scale (dichotomized for ≥3 and <3), WFNS scale (dichotomized for ≥4 and <4), type of aneurysm occlusion (microsurgical vs. endovascular) and ICP crisis (>20 mmHg) as confounders. The results are presented using regression coefficient (95% CI) the *p*-values. Abbreviations: cerebral vasospasm (CV); cerebral blood flow velocity (CBFV); confidence interval (CI); carotid siphon calcification (CSC); World Federation of Neurosurgical Societies (WFNS); intracranial pressure (ICP). * Analysis based on the sub-cohort of 121 cases with documented maximal CBFV values.

	Severity of Angiographic CV		Severity of TCD CV (Measured by the Maximum CBFV) *		Days of Increased TCD Values (>120 cm/s)	
Prediction of CV Severity	Regression Coefficients (95% CI)	*p*-Value	Regression Coefficients (95% CI)	*p*-Value	Regression Coefficients (95% CI)	*p*-Value
CSC, per-point increase	−0.14 (−0.24 to −0.04)	0.006	+1.57 (−7.45 to +10.58)	0.731	−0.43 (−0.77 to −0.10)	0.010
Patient age, per-year increase	−0.01 (−0.02 to +0.00)	0.038	−0.97 (−1.82 to −0.12)	0.026	−0.07 (−0.09 to −0.05)	<0.001
Patient sex (female)	+0.13 (−0.06 to +0.31)	0.184	+2.45 (−16.17 to +21.06)	0.795	+0.44 (−0.18 to +1.06)	0.167
Smoking status (yes)	−0.23 (−0.42 to −0.04)	0.019	−11.79 (−30.26 to +6.68)	0.208	−0.57 (−1.20 to +0.07)	0.079
Fisher grade (3 or 4)	+0.23 (−0.04 to +0.50)	0.088	+66.126 (+23.36 to 108.89)	0.003	+1.17 (+0.31 to +2.03)	0.008
WFNS grade (4 or 5)	−0.11 (−0.31 to +0.09)	0.301	−4.73 (−26.73 to +17.28)	0.671	+0.19 (−0.48 to +0.85)	0.583
Aneurysm occlusion (microsurgical)	+0.15 (−0.04 to +0.34)	0.132	−6.29 (−25.21 to +12.64)	0.511	+1.158 (+0.53 to +1.78)	<0.001
ICP crisis (>20 mmHg)	+0.33 (+0.11 to +0.55)	0.003	+34.99 (+13.07 to +56.91)	0.002	+0.93 (+0.23 to 1.63)	0.009

**Table 4:** Multinominal linear regression for the prediction of the outcome 6 months after discharge, using the mRS as a continuous variable, with the following confounders: maximum CSC per patient (using the scale by Woodcock et al. [13]), severity of radiologic CV (using the scale by Merkel et al. [14]), patient age, patient sex, smoking status, Fisher scale (dichotomized for ≥3 and <3), WFNS scale (dichotomized for ≥4 and <4), type of aneurysm occlusion (microsurgical vs. endovascular) and ICP crisis (>20 mmHg) as confounders. The results are presented using regression coefficient (95% CI) and *p*-values. Abbreviations: modified ranking scale (mRS); cerebral vasospasm (CV); confidence interval (CI); carotid siphon calcification (CSC); World Federation of Neurosurgical Societies (WFNS); intracranial pressure (ICP).

Prediction of mRS 6 Months After Discharge	Regression Coefficients (95% CI)	*p*-Value
CSC, per-point increase	+0.17 (+0.11 to +0.24)	<0.001
Severity of radiologic CV	+0.12 (+0.05 to +0.18)	<0.001
Patient age, per-year increase	+0.04 (+0.03 to +0.04)	<0.001
Patient sex (female)	−0.16 (−0.28 to −0.03)	0.014
Smoking status (yes)	−0.58 (−0.71 to −0.46)	<0.001
Fisher grade (3 or 4)	+0.34 (+0.16 to +0.52)	<0.001
WFNS grade (4 or 5)	+1.23 (+1.09 to +1.37)	<0.001
Aneurysm occlusion (microsurgical)	−0.08 (−0.20 to +0.05)	0.214
ICP crisis (>20 mmHg)	+1.37 (+1.22 to +1.5)	<0.001

## Data Availability

The data will be available from the corresponding author upon reasonable request.

## Abbreviations

The following abbreviations are used in this manuscript:

CV	Cerebral vasospasm
aSAH	Aneurysmal subarachnoid hemorrhage
CSC	Carotid siphon calcification
TCD	Transcranial Doppler
DIND	Delayed ischemic neurological deficit
aOR	Adjusted odds ratio
CI	Confidence interval
RC	Regression coefficient
DCI	Delayed cerebral ischemia
CT	Computer tomography
DSA	Digital subtraction angiography
ICP	Intracranial pressure
CBFV	Cerebral blood flow velocity
WFNS	World Federation of Neurosurgical Societies
mRS	Modified ranking scale
SD	Standard deviation
ICA	Internal carotid artery
MCA	Middel cerebral artery
ACA	Anterior cerebral artery
